# PDA+: A Personal Digital Assistant for Obesity Treatment - An RCT testing the use of technology to enhance weight loss treatment for veterans

**DOI:** 10.1186/1471-2458-11-223

**Published:** 2011-04-11

**Authors:** Jennifer M Duncan, E Amy Janke, Andrea T Kozak, Megan Roehrig, Stephanie W Russell, H Gene McFadden, Andrew Demott, Alex Pictor, Don Hedeker, Bonnie Spring

**Affiliations:** 1 Center for Management of Complex Chronic Care, Hines VA Medical Center, Hines, IL,USA; 2 Department of Preventive Medicine, Northwestern University, Chicago, IL,USA; 3 Department of Behavioral and Social Sciences, University of the Sciences, Philadelphia, PA,USA; 4 Department of Psychology, Oakland University, Rochester, MI,USA; 5 Department of Epidemiology and Biostatistics, University of Illinois-Chicago, Chicago, IL,USA

## Abstract

**Background:**

Obese adults struggle to make the changes necessary to achieve even modest weight loss, though a decrease in weight by as little as 10% can have significant health benefits. Failure to meet weight loss goals may in part be associated with barriers to obesity treatment. Wide-spread dissemination of evidence-based obesity treatment faces multiple challenges including cost, access, and implementing the programmatic characteristics on a large scale.

**Aims:**

The PDA+: A Personal Digital Assistant for Obesity Treatment randomized controlled trial (RCT) was designed to test whether a PDA-based behavioral intervention enhances the effectiveness of the existing group weight loss treatment program at VA Medical Centers Managing Overweight/Obese Veterans Everywhere (*MOVE!*). We also aim to introduce technology as a way to overcome systemic barriers of traditional obesity treatment.

**Methods/Design:**

Veterans enrolled in the *MOVE! *group at the Hines Hospital VAMC with BMI ≥ 25 and ≤ 40 and weigh < 400 pounds, experience chronic pain (≥ 4 on the NRS-I scale for ≥ 6 months prior to enrollment) and are able to participate in a moderate intensity exercise program will be recruited and screened for eligibility. Participants will be randomized to receive either: a) *MOVE! *treatment alone (Standard Care) or b) Standard Care plus PDA (PDA+). Those randomized to PDA+ will record dietary intake, physical activity, and weight on the PDA. In addition, they will also record mood and pain intensity, and receive biweekly telephone support for the first 6-months of the 12-month study. All participants will attend in-person lab sessions every three months to complete questionnaires and for the collection of anthropomorphic data. Weight loss and decrease in pain level intensity are the primary outcomes.

**Discussion:**

The PDA+ trial represents an important step in understanding ways to improve the use of technology in obesity treatment. The trial will address barriers to obesity care by implementing effective behavioral components of a weight loss intervention and delivering high intensity, low cost obesity treatment. This RCT also tests an intervention approach supported by handheld technology in a population traditionally considered to have lower levels of technology literacy.

**Trial Registration:**

ClinicalTrials.gov: NCT00371462

## Background

The obesity epidemic is recognized as an increasingly significant U.S. health problem [1]. The percentage of overweight (BMI ≥ 25-29.9 kg/m^2^) and obese (BMI ≥ 30) adults has increased in the U.S. from 46% in 1960 to 74% in 2007-08 [2]. Obesity markedly increases the risk of cardiovascular disease (CVD), cancer, Type 2 diabetes, osteoarthritis, gall bladder disease, sleep apnea, respiratory disease, pain related conditions, and premature mortality [1,2]. In addition to adverse medical consequences, obesity takes a toll on quality of life. Among veterans specifically, BMI in the obese range is significantly associated with presence of self-reported pain [3]. Weight loss efforts are confounded by the comorbid effects of obesity, pain, and the aforementioned health conditions.

Guidelines recommending lifestyle modifications for all obese patients, including diet modification and increased physical activity have been in place for more than 40 years. Yet, fewer than 25% of U.S. adults maintain diet or physical activity in accord with these recommendations [4-6]. A decrease in weight by as little as 10% can have significant health benefits on obesity-related morbidity and mortality [7]. Despite this, few adults can make the changes necessary to achieve even this amount of weight loss. It remains unclear to healthcare professionals what treatment works best to generate achievable weight loss that is cost-effective and easily disseminated.

### Obesity Treatment

There is growing evidence that behavioral treatment and lifestyle interventions are effective in moderate weight loss [8]. National Institutes of Health (NIH) guidelines advise that weight loss and weight maintenance therapy should be based on a comprehensive weight management program, including changes to diet, physical activity, and behavior goal setting [9]. Supporting these guidelines, studies of lifestyle interventions indicate that the most successful determinants are those that include an educational component addressing diet and physical activity, as well as behavior therapy, incentives, and health professional follow-up [10]. It is also recommended that individualized goals be set to reduce body weight at an optimal rate of 0.5 to 1 kg per week for the first 6 months to achieve an overall 10% weight loss [9]. Behavioral counseling has assisted overweight and obese patients to achieve clinically significant (3-5 kg) weight loss that is sustainable for up to 2 years [1,9-11]. However, the treatments that have been proven to work present a number of barriers including cost, access, and large-scale implementation.

### Barriers to Obesity Treatment

The current healthcare system cannot manage the large numbers of patients who need weight loss treatment. Evidence suggests that meaningful obesity treatment must provide individuals with frequent and immediate feedback about diet, exercise, and ways to implement new behaviors in order to achieve weight loss [12]. The costs for these types of treatment are far beyond what individuals and the healthcare system can sustain [12]. U.S. obesity-attributable medical expenditures are estimated at $75 billion annually, with $17 billion financed by Medicare and $21 billion financed by Medicaid [13]. If the trend toward obesity continues, these expenditures will reach up to 16-18% of total U.S. healthcare costs by 2030, equaling $861-957 billion dollars [14]. The challenge for interventions dealing with obesity is to identify how these treatments can be integrated into everyday care in cost effective ways that are not burdensome to patients.

Pain is also considered a barrier in healthy lifestyle interventions, especially in the older population when integrating an exercise component. In the general population and among Veterans, elevated BMI is correlated with reported pain [15,16]. Obesity co-occurs with a number of chronic pain conditions including degenerative arthritis, low back pain, and musculoskeletal pain [17,18]. In combination, pain and obesity have an additive negative effect on health-related quality of life, a finding demonstrated in both patients seeking treatment for obesity[16] and patients seeking treatment for chronic pain [19]. The impact on health care expenditures is substantial because both obesity [20-22] and pain [23,24] are associated with increased care utilization. Healthy lifestyle change is therefore a shared focus of empirically validated treatments for both obesity and chronic pain. The proposed intervention was developed to address the aforementioned barriers to obesity care by delivering effective behavioral components while delivering high intensity, low cost obesity treatment.

### MOVE! Obesity Treatment

Large scale institutions such as Medicare, the National Institutes of Health (NIH), the National Centers for Prevention (NCP), and the Veterans Affairs Medical Centers (VAMC) have all recognized the impact of the obesity epidemic and have developed community-based treatment interventions. One such development is the nationally based Managing Obese Veterans Everywhere *(MOVE!) *program offered at VA medical centers. *MOVE! *is a response to the obesity crisis affecting the veteran population at a significantly higher rate (73% males and 68% females) than the general population (67% males, 62% females) [25]. *MOVE! *is a stepped care program for obesity treatment, using 5-level treatment model. Level 1 implements self-assessment (*MOVE! 23 *questionnaire) and guided self-care. Level 2, targeted for this study, addresses patients who have indicated to their primary care physician or dieticians that they are ready to make behavioral changes in diet and physical activity. Patients receiving Level 2 treatment utilize the Level 1 assessment and self-care strategies, but also participate in group sessions and consultation addressing nutrition, physical activity, and behavior change. *MOVE! *Levels 3 - 5 involve pharmacological, inpatient, and surgical treatments respectively. Levels 1-2 are the most widely implemented delivery formats of *MOVE!*; however they present some practical barriers for those requiring treatment. Veterans identify barriers to participation that includes the time of day the program is offered, and an inability to travel to the hospitals where the program is offered. One potential way to overcome these obstacles is to introduce novel technology that enables cheaper, distance participation and swifter response times as part of obesity treatment.

### Improving Obesity Treatment with Handheld Technology

Self-monitoring has emerged as a critical skill for obesity management [26-28]. Those who report monitoring their weight on a daily or weekly basis have greater success in achieving weight loss goals [7,29-31]. Several studies have shown that PDAs are a reliable tool for dietary self-monitoring and have improved compliance and health indicators (i.e. dietary intake, glucose monitoring, blood pressure) [12,32-34] In spite of these findings, research on the use of handheld technology for self-monitoring behavior, and the impact of integrating a PDA on weight loss into behavioral weight control programs is limited [35]. Handheld technology holds great promise as a mechanism for supporting and disseminating behavioral interventions because they can deliver tailored messages at the point of decision-making in response to the needs of the user at that moment [36,37].

The PDA platform we have developed automatically codes dietary data, and has algorithms that immediately provide real-time feedback about calorie consumption and daily physical activity. Recording dietary intake and activity onto a PDA can motivate timely reporting namely because the user receives immediate feedback relative to their goals. Our technology circumvents the need for pencil and paper reports, which is cumbersome because it requires patients to carry and record onto forms, is prone to significant inaccuracies, and is not subject to timely feedback [26,38]. Another advantage to using handheld technology is that the safety of distance communication may enable patients to feel less self-conscious and freer to report accurately and communicate openly [12,26,33,38]. Installing decision-support tools on the PDA reduces the costs associated with face-to-face meetings with professionals and encourages the regular practice necessary to maintain skill acquisition over time. As a delivery vehicle, the PDA is convenient, removes access barriers, allows tailoring, and disseminates knowledge and expertise in a manner that empowers patients. Our study will implement a PDA that can be integrated into existing care, which may substantially increase the accessibility of an intervention while eliminating the costs associated with intensive weight loss treatment. What is particularly exciting about our study is that we hope to develop a modularized treatment tool that can be independently introduced to programs to increase efficacy and decrease costs.

### Study Aims

The primary aims of the proposed study are to determine whether overweight and obese patients with chronic pain who are randomized to the Standard Care + PDA group (PDA+) show more weight loss over a 6-month period, greater maintenance of weight loss at 12 months, and greater reduction in pain intensity and pain-related disability than those randomized to receive Standard Care only. We hypothesize that the addition of a PDA weight loss decision-support tool for patients plus personalized coaching will improve weight loss, weight loss maintenance, and pain reduction for obese patients with chronic pain, as compared to *MOVE! *standard care.

The secondary aims are to determine whether overweight/obese patients with chronic pain who are randomized to the PDA+ group will show significantly improved quality of life, greater treatment adherence, and reduced care utilization as compared to Standard Care only.

Secondary hypotheses are that the addition of a PDA weight loss decision-support tool for patients plus personalized coaching will improve quality of life and treatment adherence and will reduce health care utilization, as compared to *MOVE! *standard care alone. Ancillary outcomes that will be collected are patient satisfaction, mood, and waist circumference.

## Methods

### Study Design

**Figure 1:**
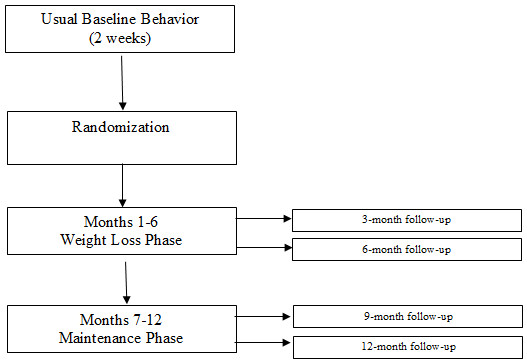
**PDA+ Trial Phases**.

The research design is a 2 × 5 factorial involving one between subject's factor (treatment condition) with 2 levels (Standard Care vs. Standard Care+ PDA (PDA+) and one within subject's factor (time) with 5 levels (randomization, 3-, 6-, 9-, and 12-month follow-up). Participants will be followed through three trial phases (Figure 1).

#### Description of Study Technology

### PDA Technology

Existing diet and activity monitoring technology on the PDA platform is limited. No other available software packages that we know of are theory based, personalized, and can be integrated into pre-existing weight loss programs. Those that are currently in use do not have sufficient empirical support to adapt to a large scale RCT as the one proposed. While the automaticity of feedback is a component of existing technology [7,32], the personalized nature of the daily feedback promises to deliver a more potent intervention.

The PDA tool, a modified version of handheld technology used in the Making Better Choices Study [39], is a patient centered decision-support system that promotes weight loss by helping participants to self-regulate eating and physical activity behaviors. The food database (used for the Nutritionist Pro Nutritional analysis program) contains accurate, up-to-date food and nutrient data for more than 18,000 foods, including brand-name foods, fast foods, and ethnic foods. The PDA program we developed has a user-friendly interface that enables participants to enter food intake and activity information. By converting complex dietary and activity data into two simple energy intake and expenditure thermometers, the PDA system will function as a decision support tool to help people track and make decisions about their momentary behaviors in relation to daily goals. Participants in the PDA+ group will also be oriented to the customizable feedback "thermometers" visually depicting how much of the day's calorie allowance has been used up and what proportion of the day's physical activity goal has been attained thus far.

### Accelerometer Technology

Participants randomized to the PDA+ group will be given an accelerometer (Biotrainer II, IM Systems, Baltimore, MD) to be worn on the hip. This triaxial accelerometer tracks moderate intensity activity by the number of counts displayed, which will provide the study team and participants with an accurate objective measure of physical activity achieved (http://www.imsystems.net/btpro/btpro.htm). The Biotrainer II has been shown to automatically detect changes in physical activity intensity, which is much more accurate than self-report [40]. The Biotrainer II will be individually calibrated for each participant, by walking on a treadmill for five minutes to demonstrate moderate intensity activity to the participant, and to orient the participant to the device display. Participants in the PDA+ group will be instructed to wear the accelerometer at all times except when immersed in water or sleeping, and re-zero the display reading at the end of each day after entry into the PDA.

### Data Collection

Data will be collected at various time points beginning at baseline and continuing through 12-month follow-up on continuous and dichotomous variables using the measures [41-50] in Table 1. Anthropomorphic measures of weight, height and waist circumference will be collected at each in-person session. Participants in the PDA+ group will remotely upload diet, physical activity, pain and mood data to their coach weekly. Data from the accelerometer will be collected at follow-up appointments. Participants will be paid $20 at randomization and at each in-person follow-up session to create an incentive that encourages retention (Total $100).

**Table 1:** Measures

Measures	Baseline	3-month	6-month	9-months	12-months
**Stage of Change**					
**Exercise Stage of Change Short Form**	x	x	x	x	x
**Weight Loss Stage of Change Short Form**	x	x	x	x	x
**Readiness to Change Pain Question**	x	x	x	x	x
**Pain Stage of Change Questionnaire (PSOCQ)**	x		x		x
**Psychological Variables**					
**PRIME-MD**	x				
**Breath Holding Task**	x				
**West Haven-Yale Multidimensional Pain Inventory (WHYMPI)**	x	x	x	x	x
**Distress Tolerance Scale**	x	x	x	x	x
**Numeric Scale of Pain Intensity (NRS-I)**	x				
**Quality of Life (SF-36)**	x	x	x	x	x
**Positive and Negative Affect Schedule (PANAS)**	x	x	x	x	x
**Other Variables**					
**VHA Patient Satisfaction Survey**			x		x
**Computer Email Web Literacy (CEW)**	x				

### Recruitment and Screening Procedures

The study procedures are approved by the Institutional Review Board at the Hines VA Medical Center. Participants who pass initial eligibility screening, will then undergo a written informed consent process prior to beginning the study protocol. To be eligible, veterans must be enrolled in the *MOVE! *group at the Hines Hospital VAMC with BMI ≥ 25 and ≤ 40 and weigh < 400 pounds, experience chronic pain ( ≥ 4 on the NRS-I scale for ≥ 6 months prior to enrollment) and are able to participate in a moderate intensity exercise program will be recruited. The enrollment policy is consistent with the 2005 VA/DoD Practice Guideline that recommends obesity treatment for all Veterans with BMI ≥ 25 and consideration of supplemental pharmacotherapy and surgical treatments for severely obese patients with BMI ≥ 40 [51]. Further eligibility criteria is listed in Table 2.

**Table 2:** Eligibility Criteria

Eligibility Criteria
• Willing to participate in a 12-month study
• Independently Mobile (no cane, walker, wheelchair needed)
• Can participate in regular exercise
• No psychiatric hospitalizations within the past year
• No cognitive or sensorimotor impairment
• Cannot participate in a structured diet program outside of study
• Cannot be at high risk for adverse CVD events while exercising
• Considering making changes to lose weight in the next 6 months^1^
• Absence of any substance abuse, mood, or binge eating disorder that would interfere with adherence^2^
• Must experience chronic pain (≥ 4 on the NRS-I scale for ≥ 6 months prior to enrollment)

^1^As measured by the Exercise and Weight Stages of Change Form

^2^As measured by the PRIME-MD

### Training

All eligible participants will receive and be trained to properly use the PDA to report dietary intake (that is automatically time and date stamped), moderate intensity physical activity minutes, and weight from a digital scale to the coach. An exclusionary competency check is administered to ensure that the participant can accurately enter a sample meal, minutes of physical activity, and their weight into the PDA. Once demonstrated, the participants will begin recording baseline data and will be scheduled for a randomization session in 7-14 days.

### Baseline Period

Baseline is a two week period in which the participant records food intake, weight, and activity into the PDA and uploads data to the study server each night. During the baseline period, participants will be required to meet the following eligibility criteria: a) record ≥ 2 meals per day and have ≥ 2 items per meal recorded, and b) record their daily weight in the morning. Coaches will provide corrective feedback when implausible reports suggest inaccurate or untimely recording (i.e: only 500 calories consumed the entire day). During the baseline phase, the intake and expenditure thermometers will not be visible to the participants. Randomization will occur if all eligibility criteria are met.

### Randomization

Prior to randomization and to discourage heightened attrition in the control group, a brief motivational interviewing intervention will be performed to make salient the pros and cons of being randomized to either condition. Participants will be re-assessed about their readiness to make lifestyle changes by administering the Exercise and Weight Stages of Change Short Forms [42] to identify and exclude pre-contemplators.

Participants will be randomly assigned to one of two groups: a) Standard Care or b) Standard Care +PDA (PDA+). Group randomization will be computer-generated via the method of randomly permuted blocks. The use of permuted blocks preserves balance in the randomization over the course of the study, to maintain equal sample sizes in the two experimental conditions. Randomization will be stratified by age (≤ 65 vs. > 65), BMI (< 35 vs. ≥ 35), and gender, to maintain equal numbers of women and men in each treatment condition.

Once randomized, those in the standard care group will no longer record their intake, weight, or activity using the PDA. The group will return all study equipment and attend *MOVE! *groups and follow-up lab sessions as scheduled.

Since the proposed design is additive (i.e., it tests the effects of adding technology and coaching to standard care), it is important to establish that the standard care received by participants is equivalent across conditions (e.g., entails comparable intervention components and level of interventionist expertise). To prevent contamination, *MOVE! *group leaders will be different individuals than those who train and coach participants randomized to the PDA conditions.

## Weight Loss Phase (months 1-6)

### Calorie and Weight Loss Goals

**Figure 2:**
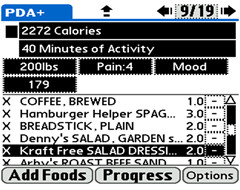
**Screen shot of the goal thermometers**. The goal thermometers reflect the calories and moderate activity of a hypothetical participant.

**Figure 3:**
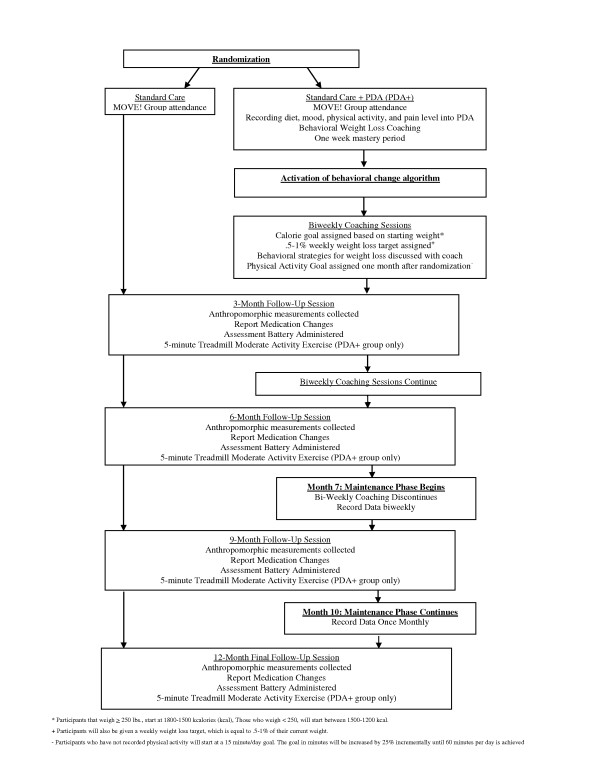
**Protocol Timeline**. Participant progression through the study protocol

Patients randomized to the treatment group will continue to upload their dietary data daily, as well as their mood, pain level, and accelerometer data (Figure 2). Successful completion of 7 days of PDA uploading meets the mastery criterion for recording and uploading, and will trigger activation of the PDA's treatment protocol (Figure 3). Rather than progressing through eating goals in a lockstep manner at fixed time intervals, progress will be mastery-based (triggered by accomplishment of each prior goal), which will help to minimize frustration and failure and reinforce intrinsic motivation.

Every two weeks for the next 6 months, coaches will review participants' data to evaluate if they have met their weight loss, calorie, and behavioral goals. If, after meeting their caloric goals, participants do not lose weight for two consecutive weeks, they will be instructed to reduce calories in 100 kcal increments until they reach a calorie intake level that yields a weight loss rate of .5-1% of their current weight per week. No patient will be given an intake goal below 1200 kcal per day. Conversely, if the rate of weight loss is too rapid (operationalized as weight loss of ≥ 3 pounds/week for 4 consecutive weeks), calorie intake goal will be increased in 100 kcal increments until attainment of the goal of .5-1% of current weight is lost per week for two weeks is achieved.

### Physical Activity Goals

Once participants have been provided with calorie intake goals for four weeks, a physical activity goal will be added. Coaches will assist participants in determining the types of activities that are ideal, which are based on interest, skill, and conditioning level. Physical activity goals are calculated and assigned using algorithms that set the treatment goals in a stepwise fashion, such that forward progression is mastery-based (i.e., patient proceeds to next goal only after first goal has been met).

## Maintenance Phase (months 7-12)

During the maintenance phase, the focus is to maintain goals set during the weight loss phase of the study. Participants are required to upload two weeks per month in months 7-9, and one week per month in months 10-12. The coach will monitor data uploads and remind participants to record and upload data if it is not completed during the pre-designated time points.

## Data Analysis

### Sample Size & Power Estimate

Sample size calculations were determined using a large VA sample provided by National Center for Health Promotion and Disease Prevention (NCP) to estimate weight loss achieved by *MOVE! *participants during a 6-month period. VA NCP data show that *MOVE! *patients who attended at least one group visit showed a weight loss of 6.36 lbs (s.d. = 11.68; N = 166) over 6 months [52]. Additionally, these same subjects had a correlation of .977 between their baseline and 6-month follow-up weight measurements. Accordingly, we expect the Standard Care group's weight loss to be 6 lb (s.d. = 12) over 6 months, and we conservatively assume this same weight loss level to be maintained at 12 months. A clinically meaningful increment in weight loss would occur if adding the PDA decision support tool produced a 12 lb. weight loss. Thus, we posit a 6 lb difference between our control and intervention groups.

To calculate power in terms of a comparison of weight measurements over time, we used methods described in Hedeker et al [53]. For this, we assume that there will be 75 participants per group at baseline, and an attrition rate of 4% at each measurement wave (i.e., an overall retention rate of 85% at 12-months). We also conservatively assume, based on the aforementioned VA study, that the correlation of the repeated weight measurements equals .95. Given these assumptions, power is in excess of .80 for a two-tailed .05 hypothesis test for the standard care versus PDA+ group contrast comparing baseline weight versus weight at all follow-up measurements (i.e., 3-, 6-, 9-, and 12-month follow-ups) under the following scenario: a 0 lb group difference at baseline and a 6 lb group difference at all subsequent timepoints (i.e., an effect size of 6/50 = .12 at these four follow-up timepoints).

For comparing weight at specific timepoints, we considered power in terms of weight change at the final timepoint, since it is at that timepoint that the sample is smallest. At 12 months, we expect there to be 75 × .85 = 64 subjects per group. If we again assume a clinically meaningful difference of 6 pounds, and given the standard deviation of 12 for weight change from the VA study, we are interested in an effect size of .5 SD units. For this, power is in excess of .80 for a two-tailed .05 hypothesis test for this comparison at the final timepoint.

### Analysis

Outcomes will be analyzed longitudinally on an intent-to-treat basis. Our general analytic approach will be to use longitudinal mixed-effects regression models (MRMs) implemented via SAS PROC MIXED. A priori contrasts will be estimated for the standard care versus PDA+ comparison. We will also include all stratification variables (age, BMI, gender) in all analyses.

Following the guidelines of Fitzmaurice and colleagues (2004) we will model weight (in lbs) at all timepoints (baseline, 3-, 6-, 9, and 12-months) including both linear and quadratic effects of time (to allow for curvilinearity in weight across time), and group by time interactions [54]. As these authors recommend, we will include baseline weight as part of the dependent variable vector, and further assume that the group means are equal at baseline. This approach yields a valid estimate of treatment group comparisons that is generally more powerful than other alternatives. Our primary tests will be for the presence of the group by time interaction terms, which will indicate the degree to which group differences in weight are present after baseline, and the degree to which any group differences are maintained over time

Though MRMs permit missing data across time, they do assume that the missing data are missing at random (MAR). MAR allows the missingness to be related to covariates (i.e., time, group, initial BMI) and also observed values of the dependent variable across time (i.e., a subject's observed weight measurements). Since many experts advise that MAR analysis is reasonable for most longitudinal clinical trials, we will use our MRM analysis as the primary approach [54,55]. However, we will also conduct sensitivity analyses to examine how robust our results are to missing data aspects using models that go beyond MAR. Specifically, we will additionally estimate pattern-mixture models [56] and shared parameter or selection models [57] to give us a sense if our conclusions vary at all by assumptions regarding the missing data.

## Discussion

The proposed research is significant in that it will test a cutting edge intervention to treat obesity. For the VA, the intervention described interfaces with existing *MOVE! *obesity programming, but aims to extend its reach and effectiveness. In addition to addressing obesity, the intervention secondarily targets pain, thus addressing two prevalent and significant clinical conditions for the VA. The research seeks to improve Veterans' quality of life by using innovative health technology in the form of a promising, non-invasive treatment that can be employed quickly and in a cost-effective manner throughout the VA system. This intervention is particularly cutting edge because it will investigate technology use in an older, predominantly male sample. Interventions such as this one will advance the understanding of older male weight loss patterns, as well as the development of effective programming to decrease the risk for obesity related comorbidities.

Technology based interventions offer scalable mechanism that may increase the scope and effectiveness of obesity treatment. Evidence suggests that technology based interventions produce better weight loss results [7,12,27,29,32,58], and increased adherence during the course of treatment [12,26,32,35]. The treatment provided in this study will build upon the strengths of technology based intervention as well as examine emerging technologies that make weight loss programs more accessible to a wide range of patients who may be less technologically savvy. The strength of this intervention is that it will integrate self-monitoring, timely feedback, regular weight monitoring, nutrition education, and behavioral weight loss strategies--all components of successful weight loss treatments.

The design of PDA+ will allow us to evaluate several hypotheses. We predict that the addition of a weight-loss decision support tool plus personalized coaching will result in greater weight loss at 6- and 12- months, as well as greater maintenance at 12-months. We also predict that the weight loss outcome will in turn affect subjective measures of pain, thus increasing quality of life outcomes. What is unclear is how these tools are disseminated into the health care system, as well as if outcomes are affected as significantly when implemented at as part of a pre-existing weight loss program.

The limitations of the generalizability of the study results warrant consideration. The population in which the intervention is being studied may not be representative of the population-at-large. Veterans have access to the *MOVE! *Group, which is a highly specialized program, and is not offered to all health care consumers. Another consideration is that the older population may have increased motivation to lose weight due to a higher incidence of health problems that would benefit from weight loss, as well as increased time per day to record into the palm program. However, given the obesity epidemic rates in the general population, the incidence of comorbid conditions will likely motivate increased weight loss efforts globally. The technology as a whole is expensive, and may not be accessible to all populations or reimbursable or accepted by public consumers. Further research is needed to specify the components of an intervention that can be scaled down to make the intervention more cost-effective. Design considerations must also be warranted in that the control group will have baseline exposure to the PDA device. The exposure may lead our control group to differ from those who have no exposure to the technology.

In sum, the PDA+ study represents an important step in understanding how to improve the use of technology in obesity treatment. The trial will answer the questions of how to provide lower-cost, effective, and efficient treatment, something that the VA continues to identify as an important component of providing care to a large number of patients. Considering that the obesity epidemic is not losing momentum, this suggests the importance of this trial that tests improved strategies to treat overweight and obese patients.

## Competing interests

The authors declare that they have no competing interests.

## Authors' contributions

BS, the principal investigator, was responsible for study conceptualization and design. She collaborated with DH, EAJ, and ATK on developing the analytic plan. JD, SWR, AD, and AP participated in operationalizing the intervention. HGM developed the data management plan. JD, BS, EAJ, ATK, MR, and SWR drafted the manuscript. All authors read and approved the final manuscript.

## Pre-publication history

The pre-publication history for this paper can be accessed here:

http://www.biomedcentral.com/1471-2458/11/223/prepub
